# Unraveling the intricacies of glioblastoma progression and recurrence: insights into the role of NFYB and oxidative phosphorylation at the single-cell level

**DOI:** 10.3389/fimmu.2024.1368685

**Published:** 2024-03-06

**Authors:** Pulin Liu, Naifei Xing, Zhikai Xiahou, Jingwei Yan, Zhiheng Lin, Junlong Zhang

**Affiliations:** ^1^ Shandong University of Traditional Chinese Medicine, Jinan, China; ^2^ Shanxi Key Laboratory of Chinese Medicine Encephalopathy, Shanxi University of Chinese Medicine, Jinzhong, China; ^3^ National International Joint Research Center of Molecular Traditional Chinese Medicine, Shanxi University of Chinese Medicine, Jinzhong, China; ^4^ Yantai Affiliated Hospital of Binzhou Medical University, Yantai, China; ^5^ China Institute of Sport and Health Science, Beijing Sport University, Beijing, China

**Keywords:** GBM, single-cell transcriptomic analysis, oxidative phosphorylation, NFYB, prognosis

## Abstract

**Background:**

Glioblastoma (GBM), with its high recurrence and mortality rates, makes it the deadliest neurological malignancy. Oxidative phosphorylation is a highly active cellular pathway in GBM, and NFYB is a tumor-associated transcription factor. Both are related to mitochondrial function, but studies on their relationship with GBM at the single-cell level are still scarce.

**Methods:**

We re-analyzed the single-cell profiles of GBM from patients with different subtypes by single-cell transcriptomic analysis and further subdivided the large population of Glioma cells into different subpopulations, explored the interrelationships and active pathways among cell stages and clinical subtypes of the populations, and investigated the relationship between the transcription factor NFYB of the key subpopulations and GBM, searching for the prognostic genes of GBM related to NFYB, and verified by experiments.

**Results:**

Glioma cells and their C5 subpopulation had the highest percentage of G2M staging and rGBM, which we hypothesized might be related to the higher dividing and proliferating ability of both Glioma and C5 subpopulations. Oxidative phosphorylation pathway activity is elevated in both the Glioma and C5 subgroup, and NFYB is a key transcription factor for the C5 subgroup, suggesting its possible involvement in GBM proliferation and recurrence, and its close association with mitochondrial function. We also identified 13 prognostic genes associated with NFYB, of which MEM60 may cause GBM patients to have a poor prognosis by promoting GBM proliferation and drug resistance. Knockdown of the NFYB was found to contribute to the inhibition of proliferation, invasion, and migration of GBM cells.

**Conclusion:**

These findings help to elucidate the key mechanisms of mitochondrial function in GBM progression and recurrence, and to establish a new prognostic model and therapeutic target based on NFYB.

## Introduction

Glioblastoma (GBM) is the most common primary intracranial malignant tumor (1) and is one of the deadliest malignant tumors due to its strong invasive and value-adding ability, coupled with its resistance to treatment, which results in most of them being able to invade the brain parenchyma and grow (2). The clinical subtypes of GBM can be categorized into low-grade glioblastoma (LGG), newly diagnosed glioblastoma (ndGBM), and recurrent glioblastoma (rGBM). Among these, LGG is an inert precursor of GBM, which usually remains unchanged for a long time and its prognosis is better proofread (3–6). In contrast, ndGBM and rGBM have a relatively poor prognosis (7, 8). Currently, multimodal therapies, such as maximal surgical resection, radiotherapy, and temozolomide adjuvant chemotherapy, are considered the primary treatment modalities for GBM. Although certain emerging therapies, including targeted therapies and tumor-treating fields, are gradually being integrated into GBM management (9–11), the complex regulatory networks of GBM, along with the tumor’s intrinsic drug-resistant phenotypes and immunosuppressive microenvironment, pose challenges for achieving sustained clinical efficacy with these novel treatment modalities (12, 13). However, currently there is no significant improvement in the prognosis of GBM patients in the last decade, with a mean survival of 12–18 months, a 1-year survival rate of 40%, and a 5-year survival rate of 5.6% (10, 14). Unfortunately, despite the current standard of care, the recurrence rate of GBM is consistently high, and once recurrence occurs, not only is drug resistance elevated and treatment options limited, but the median survival rate drops to less than 9 months (15–18). Therefore, it is particularly important to explore the mechanisms involved in the value-added and invasive ability of GBM, to search for the possible causes of GBM recurrence, and to investigate new therapeutic options and prognostic models. Currently, there is relevant research on immune therapy and related genetic pathways in GBM (19, 20). However, studies investigating the oxidative phosphorylation and transcription factor-related functions of GBM at the single-cell level are still lacking.

Redox homeostasis is the basis for maintaining normal physiological function and survival of cells (21), and oxidative processes in tumor cells tend to be more intense than in normal cells (22). These oxidative reactions are closely related to mitochondria, where oxidative phosphorylation occurs in the mitochondrial matrix, and are associated with the progression of various cancers, and some products such as oxygen-containing molecules—reactive oxygen species (ROS) (23–25)—have the potential to damage DNA and increase the risk of cancer (26, 27). In contrast, transcription factors are present in large quantities in cells and specifically regulate gene expression through different signaling pathways, thus regulating and influencing various physiological activities of cells (28). The relationship between transcription factors and tumors is very close; i.e., they can inhibit the formation and progression of tumors, such as p53 and FOXO3a (29, 30), and they can also deregulate and alter the signal transduction in important signaling pathways, so as to make the cell division and proliferation out of control, thus promoting the progression of tumors, such as the well-known NF-κB (31, 32), and can also enable tumor drug resistance by activating anti-apoptotic genes (33) and lead to poor clinical prognosis of cancer patients. The oxidative phosphorylation process occurring in mitochondria can be regulated by the interaction between nuclear transcription factors and mitochondrial proteins, as well as the binding of regulatory elements in mitochondrial DNA (34, 35). Recent literature has also revealed the regulatory role of transcription factors in oxidative phosphorylation processes in tumors. For instance, Zhao et al. discovered that the transcription factor FXR activates DHRS9 to inhibit cellular oxidative phosphorylation and suppress the progression of colon cancer (36). Given the poor prognosis and invasive recurrence of GBM, it is crucial to delve deeply into the specific mechanisms of GBM, study the specific roles of relevant transcription factors in GBM, and identify new therapeutic targets and approaches. These efforts are essential for improving treatment outcomes for GBM patients and establishing innovative prognostic models.

In this study, we conducted comprehensive research at the single-cell level to elucidate the mechanisms underlying various clinical subtypes of GBM. Employing a subtyping approach to study Glioma, we delineated cellular trajectories and enriched functions within relevant subgroups and explored the transcription factors of key subgroups. Based on these findings, we hypothesized a strong correlation between the progression of GBM and the oxidative phosphorylation pathway, as well as the transcription factor NF-YB, which was confirmed through our validated evidence. These studies contribute to a deeper understanding of GBM recurrence and progression at the cellular level, aiming to provide insights for the design of better treatment targets and prognostic models for GBM.

## Methods

### Downloading and processing of relevant data

Data from one or more tumor regions of GBM patients (GSE182109) were searched and downloaded through the NCBI Gene Expression Omnibus (GEO) database (https://www.ncbi.nlm.nih.gov/geo/), which contains a total of 44 single-cell data samples of GBM (GSM5518596-GSM5518639). In The Cancer Genome Atlas (TCGA) database (https://portal.gdc.cancer.gov) to download gene expression quantification RNA-Seq and clinical data from GBM and normalized their data using R software (R 4.3.0).

### Quality control

The samples were processed using the R package DoubletFinder to filter out and remove doublets at the cellular level (37, 38). Subsequently, a further filtering step was performed to eliminate low-quality data based on the following criteria: 1,200 < total number of genes detected in a single cell (nFeature) < 6,000; 2,500 < total transcriptomic count in a single cell (nCount) < 8,000; mitochondrial gene proportion in a single cell < 25%; red blood cell gene proportion in a single cell < 5%.

### Downscaling clustering and annotation process

Normalize the filtered single-cell data using the NormalizeData function of the R package Seurat (39, 40), calculate the variance of each gene through the FindVariableFeatures function, and screen the top 2,000 highly variable genes from the gene expression matrix based on the degree of gene dispersion and average expression (41). Continue to standardize and center all genes by ScaleData function and get rid of variant data. The CellCycleFeatures function was utilized to calculate cell cycle scores and distribution effects. The standardized highly variable genes were subjected to principal component analysis (PCA) and downscaling of single-cell data using the RunPCA function, and the batch effect of the samples was eliminated through the R package harmony. Then, the PCA downscaled cells were clustered by the FindNeighbors function and FindClusters function in the R package Seurat. Based on previous relevant literature, the cells in different clusters were annotated by combining the Cellmarker database (http://bio-bigdata.hrbmu.edu.cn/CellMarker/) and SingleR. We investigated the sample sources of different subclusters and identified differentially expressed marker genes among clusters by the FindAllMarkers function.

### Staging and clinical subtyping of large clusters of cells

We analyzed the cellular staging among GBM cell populations and visualized them with UMAP to explore the related situation of division and proliferation of GBM cell populations (42, 43), and similarly, we followed the above method to investigate the proportion and distribution of clinical subtypes of GBM cell populations. In order to further investigate the correlation between the stages and clinical subtypes, we also calculated the G2M score and S score of different cell populations and clinical subtypes, and visualized the gene expression of each cell population of GBM by UMAP Functional analysis.

### Cell population enrichment

We identified differentially expressed genes (DEGs) in different cell populations of GBM, and the screening criteria were that DEGs were required to be detected in 25% of the cells with *p* < 0.01, false discovery rate (FDR) < 0.05, and |logFCfilter| >1, and the screened DEGs of each cell population were subjected to a Gene Ontology (GO) enrichment analysis. We also performed a Gene Set Enrichment Analysis (GSEA) by calculating the DEGs between Glioma and other clusters using the Kyoto Encyclopedia of Genes and Genomes (KEGG). The set of marker genes collected in the database (c2.cp.kegg. v7.5.1.symbols.gmt) was filtered and analyzed, defining statistical significance as FDR < 0.05.

### Metabolic analysis of large populations of cells

We calculated the cellular metabolic pathway profiles of each cell population, each stage, and each clinical subtype of GBM using the R package scMetabolism, and had the top 20 pathways visualized in the form of heatmaps. We further filtered out the top three ranked pathways, investigated their distribution in UMAP, and explored their expression in GBM macro-populations, cell staging, and clinical subtypes.

### Glioma subgroup correlation analysis

The FindNeighbors function and FindClusters function in the Seurat package were used to analyze the correlation between the Glioma cell clusters that were reclustered to calculate the chromosome copy variation number (inferCNV) of different subpopulations of Glioma, and to find the subpopulation marker genes with reference to the percentage and total amount of gene expression in each subpopulation of cells. The cellular staging and clinical subtype proportions of the cell subpopulations were calculated and visualized using UMAP. We further calculated the G2M scores of each clinical subtype for each subpopulation and counted the Glioma cell population and explored the density of subpopulation G2M scores in combination with UMAP plots of G2M scores to find and identify key subpopulations. We calculated the G2M score for Glioma scores for each subpopulation intercellular DEGs for GO and GSEA enrichment function analysis and explored the Glioma metabolic pathways of each subpopulation and each clinical subtype, and visualized them in heatmap format to explore the relevant metabolic and functional profiles of each subpopulation.

### Glioma cell subpopulation trajectory analysis

In order to understand the metabolic pathways of Glioma subpopulations, the progression of cell development and differentiation among subpopulations was analyzed in a proposed time series. The differentiation status of cell subpopulations was predicted using the R package Cytotrace, with a score interval of 0–1, and the score was positively correlated with cell stemness. We also used slingshot to predict the cell clustering and downscaling information based on the Glioma cell subpopulations to infer the genealogical structure and proposed temporal sequence, and further investigated the temporal expression of the key subpopulation C5 differentiation spectrum, cellular staging, and clinical subtypes in order to explore the relevant properties of the C5 subpopulation.

### Analysis of cell subpopulation interactions and transcription factors

We employed the R package cellchat to computationally analyze the intercellular interactions between various subpopulations of Glioma and major cell groups within GBM. By predicting the quantity and strength of interactions between cells based on the average expression levels of receptors and ligands, we visualized the different signal intensities transmitted to and from each cell type. Additionally, we calculated the cell stemness genes within each Glioma subpopulation. To investigate the top five transcription factors (TFs) that exhibited the most significant changes in expression proportions for each subpopulation, we utilized the computational approach provided by pySCENIC. Firstly, we employed GRNBoost to identify the potential target genes of each TF. Then, utilizing DNA-motif analysis, we selected the potential direct binding targets. Subsequently, we used AUcell to score the activity of regulons in cells, ultimately selecting the top five TFs with the highest scores. Finally, we explored the expression patterns of these C5-related TFs across different subpopulations.

### Clinical correlation and independent prognosis analysis of C5 subpopulations

By reviewing the literature, we found that the key transcription factor NFYB of the C5 subgroup has relatively little research literature in GBM; in order to further study its interrelationship with GBM, we extracted the target genes within the regulatory module of NFYB and took the intersection of them with the genes of the normal and tumor tissues of GBM and merged them with the filtered and standardized clinical data. Univariate Cox risk regression analysis was performed using the coxph function in the R package survival and validated by last absolute shrinkage and selection operator (LASSO)-penalized Cox regression and multivariate Cox risk regression analysis to obtain prognostic differential genes (44, 45). The risk scores of the samples were calculated (risk score = Xλ is the relative expression level of prognosis-related genes, coefλ is the coefficient) (46, 47), and the samples were divided into high- and low-risk groups according to the median, and the distribution was examined by principal component analysis (PCA). Heatmaps were used to visualize the expression of prognostic genes in the high- and low-risk groups, and the coef value of each prognostic gene was calculated. The different time survival of the high- and low-risk groups was demonstrated using Kaplan–Meier curves (48, 49), and the prognostic specificity and sensitivity were verified by time-dependent receiver operating characteristic (ROC) curves (50). We further investigated the correlation between prognostic genes and risk scores. We also calculated the correlation between age, race, and risk score in the sample and plotted a nomogram to predict the prognosis of ovarian cancer patients in conjunction with the risk score using R packet rms (51), which was validated using ROC curves and decision curve analysis (DCA).

### Immune correlation and enrichment analysis

We performed immune infiltration analysis of patients in high- and low-risk groups using the xCell and CIBERSORT deconvolution algorithms. We investigated the correlation between immune infiltration and risk scores as well as prognostic genes. Furthermore, we utilized the R package “estimate” to calculate the immune score, stromal score, tumor purity score, and overall score of the tumor microenvironment in the high- and low-expression groups. We also explored the TIDE (Tumor Immune Dysfunction and Exclusion) scores in the high- and low-risk groups. The expression patterns of immune checkpoint-related genes in the high- and low-expression groups were estimated using the Wilcoxon test and visualized in relation to risk scores and prognostic genes. Differential gene expression analysis between the high- and low-risk groups was performed using the limma package in R, with the following filtering criteria: |log2FoldChange| > 1 and FDR (BH) adjusted threshold (*p*_adj_) < 0.05. GO and Kyoto Encyclopedia of Genes and Genomes (KEGG) enrichment analysis was conducted using the “clusterProfiler” R package (52–54).

### Cell culture

Both the U87 MG cell line and the U251 MG cell line were obtained from the American Type Culture Collection (ATCC). The two cell lines were cultured in F12K medium containing 10% fetal bovine serum (Gibco BRL, USA) and 1% streptomycin/penicillin and PRMI1640 medium (Gibco BRL, USA), respectively, under standard conditions (37°C, 5% CO_2_, 95% humidity).

### Cell transfection

NFYB knockdown was achieved using small interfering RNA (siRNA) constructs (GenePharma, Suzhou, China). The transfection protocol was performed according to the steps described for Lipofectamine 3000RNAiMAX (Invitrogen, USA). Cells were inoculated in six-well plates at 50% fitness and then infected with negative control (si-NC) and knockdown (Si-NFYB-1 and Si- NFYB -2). Each transfection was performed using Lipofectamine 3000RNAiMAX (Invitrogen, USA).

### Cell viability assay

Cell viability of U87 MG cells and U251 MG cells after transfection was detected by CCK-8. The cell suspension was inoculated at a density of 5 × 10^3^ cells per well in a 96-well plate and incubated for 24 h. CCK-8 marker (10 μL; A311-01, Vazyme) was added to each well, and incubated away from light at 37°C for 2 h. Cell viability was assessed by detecting the absorbance of the enzyme marker (A33978, Thermo) at 450 nm on days 1, 2, 3, and 4, respectively. The average OD values were calculated and plotted on a line graph.

### 5-Ethyl-2'-deoxyuridine proliferation assay

Transfected U87 MG cells and U251 MG cells were inoculated in six-well plates at a density of 5 × 10^3^ cells per well and cultured overnight. A 2× EdU working solution was prepared by adding 10 mM EdU solution in serum-free medium, added to the cell culture and incubated at 37°C for 2 h. The medium was removed and washed with PBS, and the cells were fixed by adding 4% paraformaldehyde for 30 min. Cells were then treated with glycine (2 mg/mL) and 0.5% Triton X-100 for 15 min. Cells were incubated with 1 mL 1× Apollo and 1 mL 1× Hoechst 33342 for 30 min at room temperature. Cell proliferation was quantified by fluorescence microscopy.

### Wound healing

The transfected cells were inoculated in six-well plates and cultured until the cell density reached 95%. First, use a 200-μL sterile pipette tip in the cell culture wells to pass through the cell layer in a straight line, and then gently rinse the culture wells with PBS. Change the medium to continue cell culture. Photographs of the scratch at the same location at 0 and 48 h were collected and the width of the scratch was measured.

### Transwell experiment

Cells were starved in serum-free medium for 24 h before the experiment. After treatment with the addition of matrix gel (BD Biosciences, USA), the cell suspension was added to the upper chamber containing Costar and serum medium was added to the lower chamber. Cells were incubated in an incubator for 48 h. After incubation, cells were fixed with 4% paraformaldehyde and stained with crystal violet to observe the invasive ability of the cells.

## Results

### Classification of GBM macroclusters

**Figure 1:**
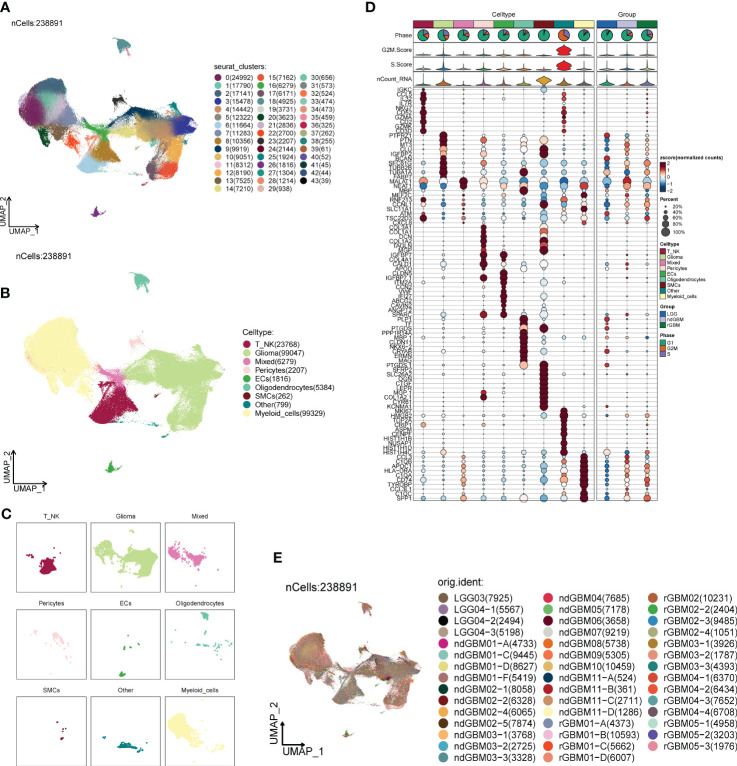
Classification of large groups of GBM cells. **(A)** A total of 44 samples from 19 GBM patients were clustered into 44 clusters. **(B)** According to different MARKER genes, the ovaries were annotated into nine cellular macrogroups of T_NK, Glioma, Mixed, Pericytes, ECs, Oligodendrocytes, SMCs, Other, and Myeloid_cells, respectively. **(C)** Distribution of cells in each cell macrocluster of GBM. **(D)** Graph of the expression of the first five MARKER genes in each macrocluster. **(E)** Distribution of the 44 sample sources.

We downloaded tumor single-cell data from one or more regions of 2 patients with LGG (LGG03 and LGG04), 11 patients with ndGBM (ndGBM01 to ndGBM11), and 5 patients with rGBM (rGBM01 to rGBM05) from the GEO database, which contained a total of 44 samples (GSM5518596–GSM5518639) (**Supplementary Figure 1A**). The doublet cells in the samples were eliminated by the R package DoubletFinder, and the samples were further filtered out of other poor-quality cells (**Supplementary Figure 1B**). We found that the distribution of cells with different staging was relatively concentrated in the PCA plots (**Supplementary Figure 1C**), indicating that the cell staging had less impact on our findings overall. We selected the top 2,000 highly variable genes (**Supplementary Figure 1D**), downscaled them using RunPCA, and intercepted the top 30 dimensions for further analysis (**Supplementary Figures 1E, F**). We also presented the top 10 highly variable genes in the top nine dimensions in a heatmap (**Supplementary Figure 1G**). Through dimensionality reduction and clustering, we categorized GBM into 44 clusters (**Figure 1A**), and further annotated and integrated the clusters into nine large groups of cells by the R package singleR and the Cellmarker database, and combined them with related literature, whose cell types and numbers were T_NK (23,768), Glioma (99,047), Mixed (6,279) Pericytes (2,207), ECs (1,816), Oligodendrocytes (5,384), Smooth Muscle Cells (SMCs) (262), Other (799), and Myeloid_cells (99,329) (**Figures 1B, C**), in which the Glioma and Myeloid_cells cell number and distribution range were larger. We also screened the top 10 marker genes of nine large clusters of cells and explored their correlations with cell staging scores and with clinical subtypes (**Figure 1D**), and we found that marker genes of Glioma and Myeloid_cells cells were highly correlated with clinical subtype-expressed genes, especially ndGBM and rGBM, and we hypothesized that these two large clusters of cells might have a greater tumor relevance. We also analyzed the sample sources of the major clusters of cells (**Figure 1E**) and found that the Glioma and Myeloid_cells cell clusters had relatively more sample sources.

### Correlation analysis of clinical subtypes and cell stages

**Figure 2:**
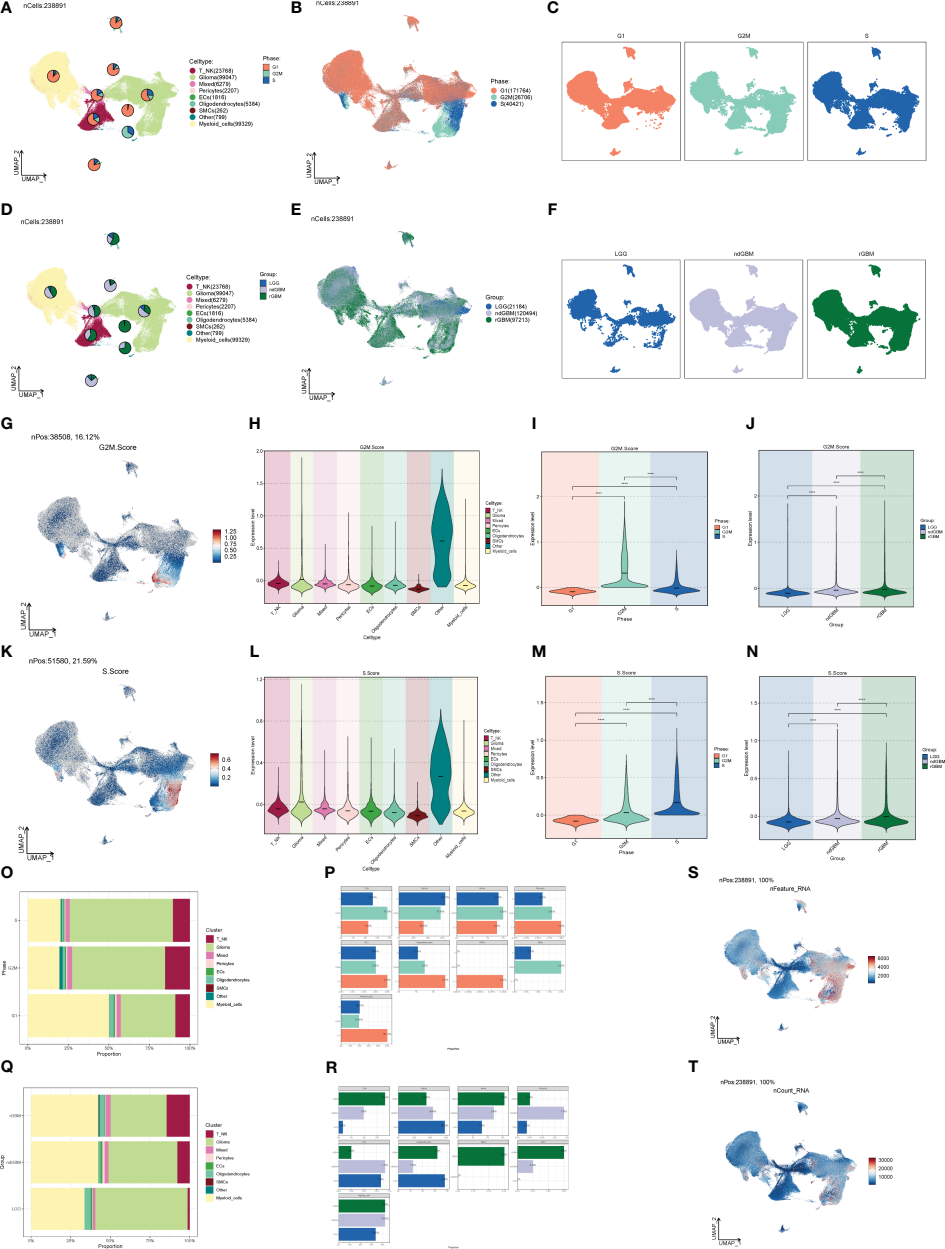
Staging and clinical subtypes of large population cells. **(A–C)** Plots of the distribution of clinical subtypes LGG, ndGBM, and rGBM in major populations of GBM cells. **(D–F)** Plots of the distribution of cell staging G2M, S, and G1 in major populations of GBM cells. **(G)** Distribution of UNMP for the G2M.Score. **(H–J)** Plots of G2M.Score violin in major populations of GBM cells, cell staging, and clinical subtypes. **(K)** UNMP distribution maps. **(L–N)** S.Score violin maps of GBM large populations of cells, cell staging, and clinical subtypes. **(O, P)** Situation maps of the proportion of cells between GBM large populations of cells and the clinical subtypes LGG, ndGBM, and rGBM. **(Q, R)** Situation maps of the proportion of cells between GBM large populations of cells and the cell staging periods G2M, S, and G1. **(S, T)** nFeature and UNMP distribution plots for nCount.

The G1 phase is the quiescent phase before DNA replication at the end of division, the S phase is the DNA replication phase, and the G2M phase involves the preparation for cell division and mitosis, and any abnormality in these phases will likely lead to tumorigenesis. Combined with cellular staging, we found that G2M was concentrated in both Glioma and Myeloid_cells (**Figures 2A–C**), in which the proportion and distribution of G2M was obviously higher than that of Myeloid_cells, but the distribution was obviously uneven, and the proportion of S was probably concentrated in one of its subpopulations. The proportion and distribution of G2M in Glioma was significantly higher than that in Myeloid_cells, but the distribution was not uniform, which might be concentrated in a certain subpopulation, and the proportion of S was the highest in all groups of cells (**Figures 2O, P**), suggesting that a certain subpopulation of Glioma had a more vigorous replicative and divisive proliferative ability. LGG, ndGBM, and rGBM represent clinical subtypes or different states of gliomas, of which LGG is a low-grade Glioma, which is slow-growing, poorly invasive, and has a good prognosis. ndGBM refers to non-enhancing glioblastoma, which also has relatively strong invasive ability and poor prognosis, while rGBM belongs to recurrent Glioma, which not only has the fastest growth and strongest invasiveness, but also tends to have the worst prognosis. Combined with the clinical subtypes of GBMs, we found that the distributions of ndGBMs and rGBMs in Glioma were highly correlated to the distribution location of G2Ms in Glioma (**Figures 2D–F**). Instead, by looking at the proportional bar graphs of the major groups of clinical subtypes (**Figures 2Q, R**), we found that the highest percentage of LGG in Glioma may be due to the overly concentrated distribution of LGG subtypes in Glioma, which is not in conflict with our finding that the distribution of ndGBM and rGBM overlapped and was concentrated in the UMAP plots, which suggests that subtyping can be continued in Glioma to identify subpopulations with higher dividing and invasive abilities. We also explored the G2M scores and S scores for each subpopulation, as well as cellular staging and clinical subtype (**Figures 2G–N**), and we found that the Glioma scores were relatively high in all but OTHER cells, and the G2M scores and S scores gradually increased in LGG, ndGBM, and rGBM, indicating a gradual increase in DNA replication and cell division capacity. We also calculated the DNA expression and replication and found that it was also concentrated in a subpopulation of Glioma (**Figures 2S, T**).

### Functional analysis of cell enrichment in large GBM populations

**Figure 3:**
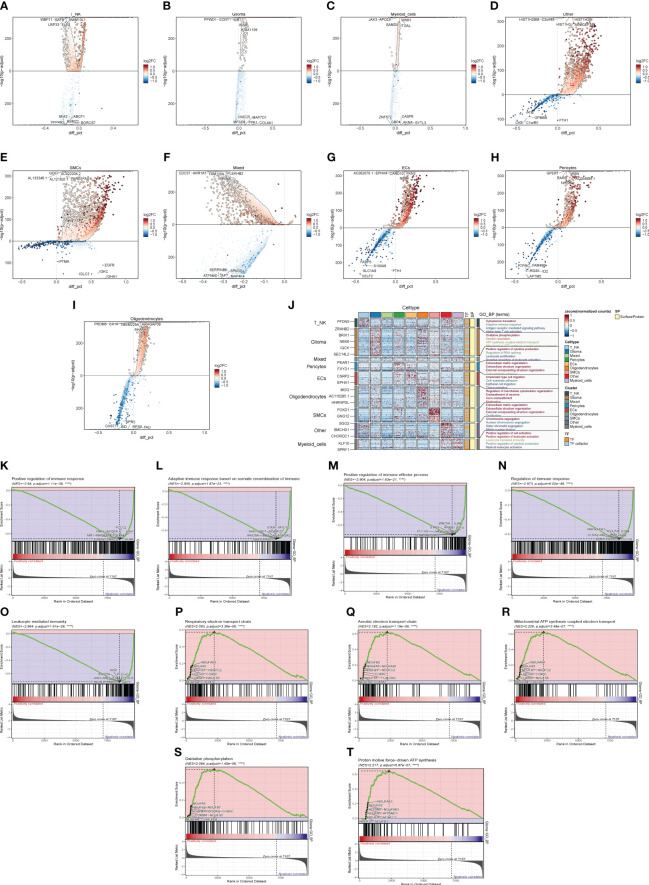
Enrichment analysis of major clusters of cells. **(A–I)** Differential gene distribution map of GBM large population cells and other cells, in which the top five selected genes from each of the high-expression and low-expression genes are shown. **(J)** GO enrichment analysis map of GBM large population cells. **(K–T)** GSEA enrichment analysis map of Glioma, in which the top five pathways from each of the low-expression group and the high-expression group were selected.

We calculated DEGs for each cell population of GBM tumor tissues and demonstrated them for the top five genes (**Figures 3A–I**). Then, we performed GO enrichment function analysis on DEGs of large cell populations (**Figure 3J**), and we found that in Glioma, the enrichment of pathways related to oxidative function was significantly increased, among which “Oxidative phosphorylation” and “Aerobic respiration” ranked high. Through GSEA enrichment analysis (**Figures 3K–T**), we found that the “Respiratory electron transport chain”, “Aerobic electron transport chain”, “Mitochondrial ATP synthesis coupled electron transport”, “Oxidative phosphorylation”, and “Proton motive force-driven ATP synthesis” pathways were significantly enriched in the high-expression group, and most of them were related to the oxidative respiration pathway, while the “Positive regulation of immune response” pathway was enriched in the high-expression group. “Positive regulation of immune response”, “Adaptive immune response based on somatic recombination of immune”, “Positive regulation of immune effector process”, “Regulation of immune response”, and “Leukocyte mediated immunity” were significantly enriched in the low-expression group, which were mostly related to immune function.

### Metabolic analysis of GBM cell populations

**Figure 4:**
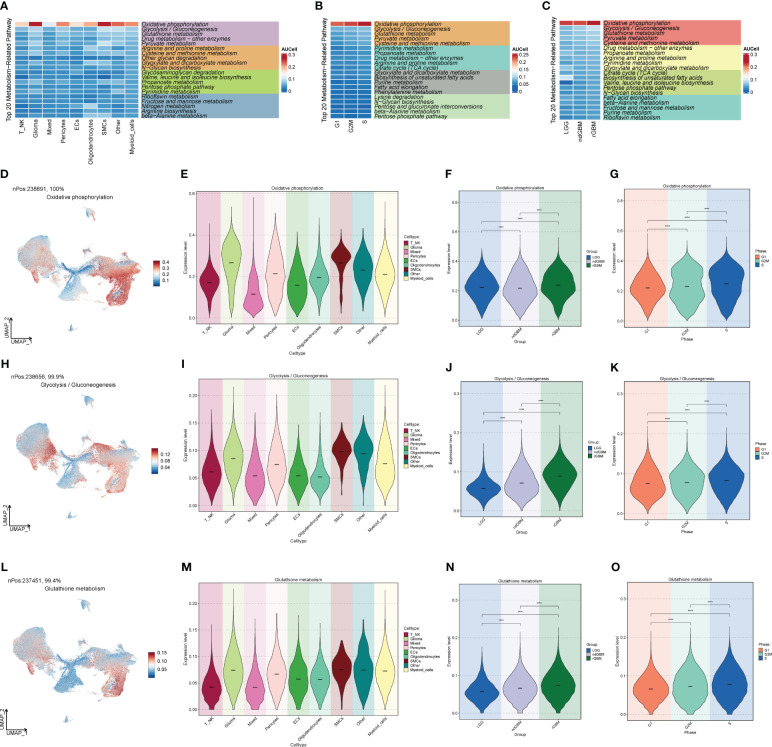
Metabolic pathway map of GBM large population cells. **(A)** Top 20 metabolic pathways of GBM large population cells. **(B)** Top 20 metabolic pathways of G2M, S, and G1 staged cells. **(C)** Top 20 metabolic pathways of LGG, ndGBM, and rGBM clinical subtypes cells. **(D–O)** “Oxidative phosphorylation”, “Glycolysis/Gluconeogenesis”, and “Glutathione metabolism” pathways and the distribution of UNMP in the GBM cell population, cell staging, and clinical subtype scores. The distribution of UNMP in GBM cells, cell staging, and clinical subtype scores were plotted. **** means p < 0.0001.

In order to study the pathways related to cellular metabolism, we calculated the metabolic pathways of GBM cell populations, cell stages, and clinical subtypes (**Figures 4A–C**), and found that the “Oxidative phosphorylation”, “Glycolysis/Gluconeogenesis”, and “Glutathione metabolism” pathways all scored high in GBM populations. “Gluconeogenesis” and “Glutathione metabolism” pathways all scored high, and their expression was further visualized in the major GBM clusters by UMAP (**Figures 4D–O**), of which the “Oxidative phosphorylation” pathway had the highest score, which is related to oxidative stress and was highly expressed in Glioma and rGBM, and thus we speculated that it might be a key pathway in tumors.

### Correlation analysis of Glioma subgroups

**Figure 5:**
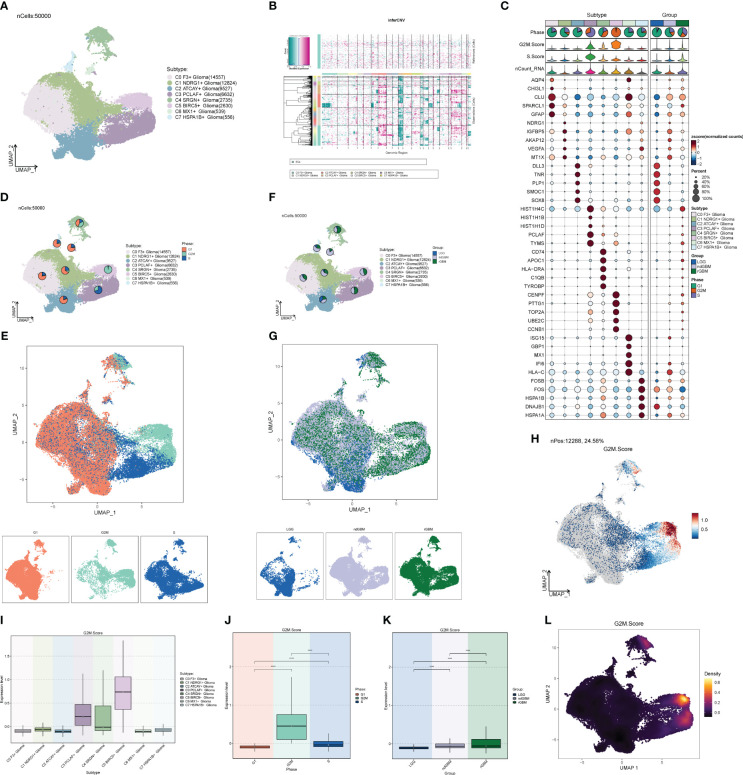
Glioma cell subpopulation and correlation analysis. **(A)** UNMP distribution of cell subpopulations. **(B)** Heatmap of CNV status of cell subpopulations. **(C)** Expression of the top five marker genes in each subpopulation of cells. **(D, E)** UNMP distribution of subpopulation of cells in each clinical subtype of LGG, ndGBM, and rGBM. **(F, G)** UNMP distribution of subpopulation of cells in each cell staging of G2M, S, and G1. **(H)** UNMP distribution of score of G2M. **(I–K)** Box plot of G2M.Score for Glioma cell subpopulations, cell staging, and clinical subtypes. **(L)** Density plot of G2M.Score for Glioma subpopulation cells. **** means p < 0.0001.

**Figure 6:**
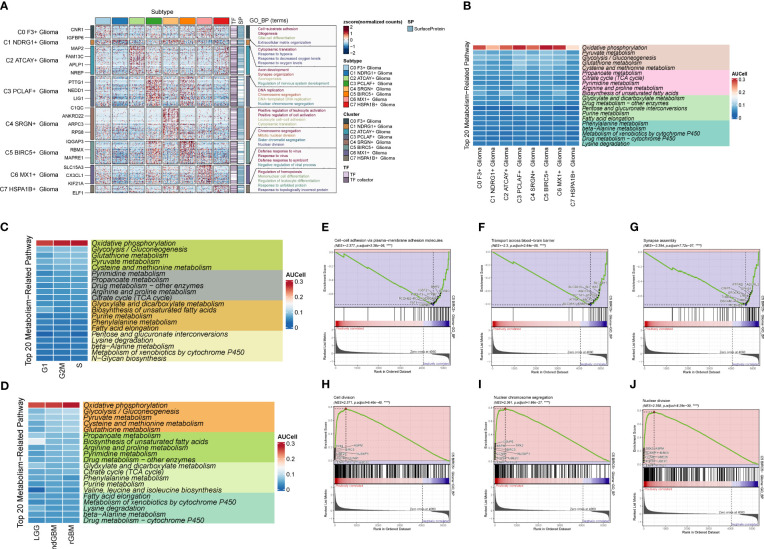
Enrichment analysis and cell metabolism analysis of Glioma cell subpopulations. **(A)** GO enrichment analysis plot of Glioma cell subpopulations. **(B)** Top 20 metabolic pathway plots of Glioma cell subpopulations. **(C)** Top 20 metabolic pathway plots of cell subpopulations of each staging cell of G2M, S, and G1. **(D)** Top 20 metabolic pathway plots of cell subpopulations of each clinical subtype of LGG, ndGBM, and rGBM. **(E–J)** GSEA enrichment analysis plots of cells of subpopulation of C5, with three cells selected from the low-expression group versus the three pathways that were selected from each of the high-expression groups.

Through G2M staging and clinical subtype analysis, we found that there were subpopulations of cells with strong dividing and proliferating ability in Glioma. Therefore, we further clustered the large Glioma population and divided it into eight cell groups, sequentially C0–C7, and selected a marker gene for each subpopulation by combining the expression and percentage of marker genes (**Figure 5A**). By calculating the CNV of each subpopulation of cells (**Figure 5B**), we found that the subpopulation of cells all had an increased mutation on chromosome 7 and a deletion of the mutation on chromosome 10. We also investigated the first 5 marker genes of each subpopulation and cell staging and clinical subtype correlation (**Figure 5C**). We then examined the proportion of cell staging in each subpopulation of cells (**Figures 5D, E**), and we found that the C5 cell subpopulation had the highest percentage of G2M, while the C3 subpopulation had the highest percentage of S staging, suggesting that this cell population had the most rapid dividing and proliferating ability, while the C3 subpopulation had the second highest. We also calculated the clinical staging of the cell subpopulations (**Figures 5F, G**) and found that the rGBM percentage was higher in the C3, C4, and C5 subpopulations, indicating that the tumor cells in these subpopulations were more aggressive and malignant. Furthermore, we investigated the G2M scores of each subpopulation and clinical subtype (**Figures 5H–L**), and found that the G2M scores of the C5 subpopulation and ndGBM and rGBM were higher, suggesting that the tumor cells of the subpopulations with high dividing ability were also relatively more invasive and malignant, which was in line with our prediction. Among these subpopulations, the c5 subpopulation had the highest G2M score and a relatively high percentage of rGBM, so we speculated that the C5 subpopulation might be associated with tumor recurrence, division, and invasiveness, and might be a key subpopulation of Glioma. By GO enrichment function analysis (**Figure 6A**), we found that the enrichment pathways of the C5 subgroup were mostly related to cell reproduction and division functions, such as “Chromosome segregation” and “Mitotic nuclear division”. The metabolic pathway analysis (**Figures 6B–D**) showed that “Oxidative phosphorylation” scored significantly higher in the key subgroups of C5 and G2M, as well as the clinical subtypes of ndGBM and rGBM, and we hypothesized that “Oxidative phosphorylation” is a key component of C5, G2M, and rGBM. We hypothesized that “Oxidative phosphorylation” may play a key role in the recurrence and progression of GBM. We also further analyzed the GSEA enrichment of the C5 subgroup (**Figures 6E–J**), and found that the pathways enriched in the high-expression group are predominantly associated with cell proliferation and replication, such as “Cell division,” “Nuclear chromosome segregation,” and “Nuclear division.” Conversely, pathways enriched in the low-expression group are associated with tumor cell migration and invasion, such as “Cell-cell adhesion via plasma-membrane adhesion molecules,” “Transport across blood-brain barrier,” and “Synapse assembly”.

### Proposed chronological analysis of GBM cell subpopulations

**Figure 7:**
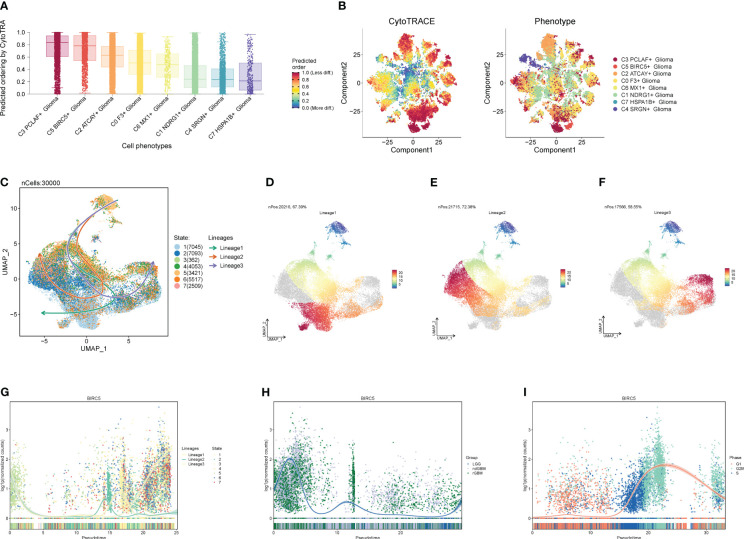
Proposed chronological analysis of cell subpopulations. **(A, B)** Cytotrace scores of cell subpopulations. **(C-F)** Slingshot-based temporal trajectory maps of cell subpopulations with three trajectory differentiation directions. **(G–I)** Trajectory changes of marker gene BIRC5 with trajectory directions, clinical subtypes, and cellular staging.

We predicted the differentiation of each subpopulation of Glioma cells using the R package Cytotrace (**Figures 7A, B**), and found that both C3 and C5 were less differentiated. To understand the cell subpopulation temporal trajectory relationship, we inferred the temporal order of the cell subpopulations by slingshot (**Figures 7C–F**) and found three trajectories in which the C5 subpopulation was located at the end of trajectory 3 and passed through the C3 subpopulation. Therefore, we hypothesized that C3 and C5 subpopulations are highly correlated and that the C5 subpopulation is the most aggressive and destructive subpopulation of the tumor. In addition, we also explored the temporal trajectory changes of BIRC5, the marker gene of subpopulation C5, with trajectory direction, clinical subtype, and cellular staging (**Figures 7G–I**), and found that it was highly overlapped with ndGBM and rGBM in the clinical subtypes and G2M in the cellular staging, which also illustrated the replicative and proliferative ability of the C5 subpopulation and the malignancy degree from the side.

### Interaction and transcription factor analysis of GBM cell subpopulations

**Figure 8:**
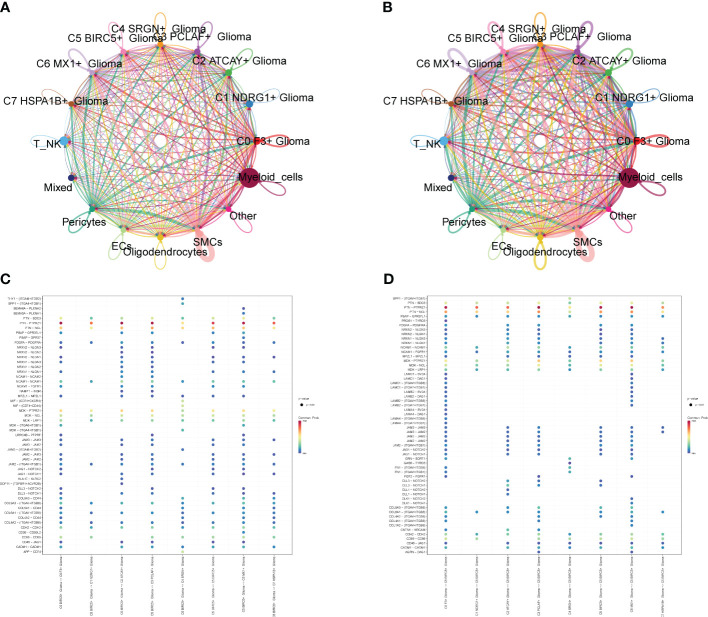
Interaction analysis between cell subpopulations and large population cells. **(A)** Plot of the number of interactions between Glioma cell subpopulations and GBM large population cells; the thicker lines represent the higher number. **(B)** Plot of the intensity of interactions between Glioma cell subpopulations and GBM large population cells; the thicker lines represent the higher intensity. **(C, D)** Plot of points of the degree of interactions between C5 subpopulation and other cell subpopulations and large population cells with receptor ligands.

**Figure 9:**
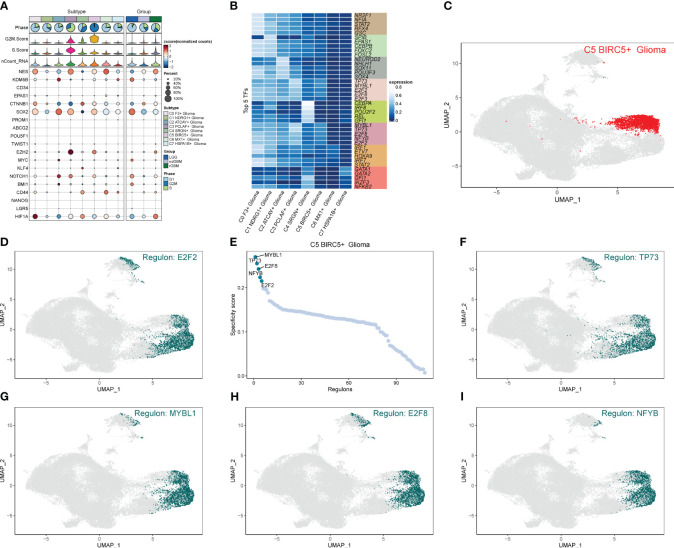
Analysis of stemness genes and transcription factors in cell subpopulations. **(A)** Expression of stemness genes in each subpopulation of Glioma. **(B)** Top five transcription factors in each subpopulation of cells in Glioma. **(C–I)** UNMP plot of C5 subpopulation of Glioma and the distribution of top five transcription factors in the subpopulation.

We calculated the strength and number of interactions between cell subpopulations and large populations of cells and visualized them with circle plots (**Figures 8A, B**), and we found that the C5 subpopulation appeared to have a higher number of interactions with large populations of SMCs, and within subpopulations, interactions were more closely related to C0 and C3. We also further investigated the receptor–ligand pairs between the C5 subpopulation and other subpopulations (**Figures 8C, D**), and we found that PTN-PTPRZ1 and PTN-NCL were more closely exchanged between C5 and the subpopulations. By studying cell stemness genes, we found that NES, SOX2, and EZH2 were more significant in C5 (**Figure 9A**). We also further investigated the transcription factors of each subpopulation (**Figure 9B**) and found that MYBL1, TP73, E2F8, NFYB, and E2F2 scored more significantly in the C5 subpopulation. We also further investigated the expression of these transcription factors in other subpopulations (**Figures 9C–I**) and found that the transcription factors in C5 were also significantly expressed in the C3 subpopulation, which, in combination with the finding that C5 and C3 were located in the same time trajectory and had higher G2M and S staging, may suggest that there is some correlation between these transcription factors and the ability to divide and proliferate.

### Prognostic correlation analysis

**Figure 10:**
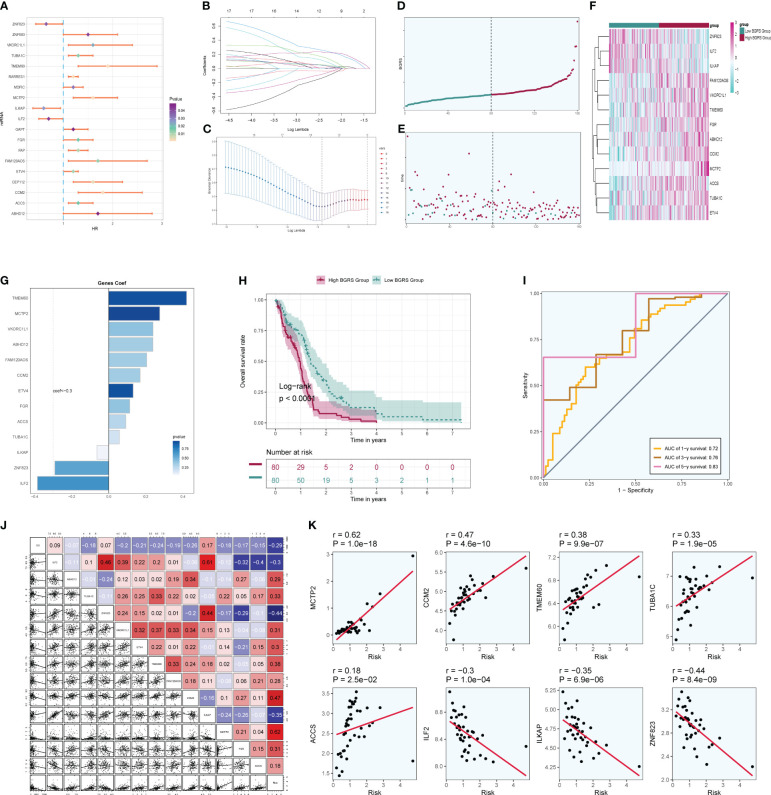
Independent prognostic analysis. **(A)** A total of 19 prognosis-related differential genes. **(B)** LASSO analysis coefficient spectrum distribution of 13 prognostic genes. **(C)** Optimal cross-validation of parameter selection in LASSO regression. **(D)** Categorization of patients into high- and low-risk groups based on risk scores. **(E)** Distribution of patients in high- and low-risk groups. **(F)** Heatmap of the distribution of prognostic-related genes. **(G)** Coef values of prognostic-related genes. **(H)** High- and low-risk groups’ Kaplan–Meier prognostic analysis curves. **(I)** Time-related ROC curves with area under the curves (AUCs) of 0.72, 0.76, and 0.83 at 1, 3, and 5 years. **(J)** Correlation analysis between genes and risk scores and OS. **(K)** Dot plots of the top eight prognostic genes that had a strong correlation with risk scores.

**Figure 11:**
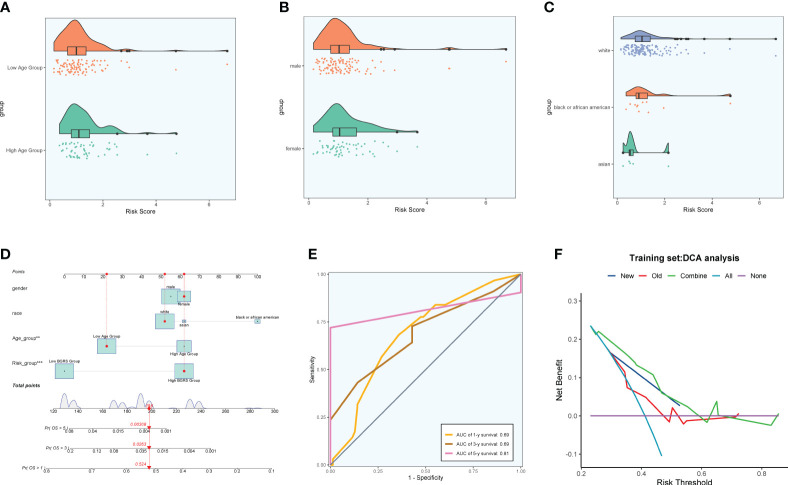
Clinical correlation analysis. **(A–C)** Analysis of the correlation between risk scores and age, gender, and ethnicity factors. **(D)** Survival column line plots of GBM patients at 1, 3, and 5 years. **(E)** Time-dependent ROC plots, with AUCs of 0.69, 0.69, and 0.81 at 1, 3, and 5 years, respectively. **(F)** DCA of prognostic models.

Firstly, NF-YB serves as a crucial transcription factor for the key subgroup C5. Secondly, there has been relatively limited literature on the relationship between NF-YB and GBM, as well as the specific mechanisms involved. Therefore, we chose to conduct further research on this transcription factor to elucidate its specific mechanisms in oxidative phosphorylation and its role in the progression of GBM, as well as its relevance in GBM prognosis. We extracted key subgroups of C5 transcription factor target genes within the NFYB regulatory module and took the intersection with the GBM differential genes downloaded from the TCGA database, and by univariate Cox risk regression analysis, we screened 19 prognostic genes associated with them (**Figure 10A**) and verified that these genes were stable and well behaved by LASSO Cox risk regression analysis (**Figures 10B, C**). Finally, 13 genes associated with prognosis were confirmed by multivariate risk Cox risk regression analysis, of which TMEM60, MCTP2, VKORC1L1, ABHD12, FAM120AOS, CCM2, ETV4, FGR, ACCS, and TUBA1C were high-risk genes, while ILKAP, ZNF823, and ILF2 were low-risk genes. We calculated the risk scores of each sample and divided them into high-risk and low-risk groups according to the median (**Figure 10D**), and we evaluated the relationship between survival status and survival time between the high- and low-risk groups (**Figure 10E**) and found that the number of patients who died significantly increased and was more centrally distributed as the risk score increased. We also examined the expression of prognostic genes in the high- and low-risk groups (**Figure 10F**) and calculated coef values (**Figure 10G**), with TMEM60 being the highest and ILF2 being the lowest. The expression of TMEM60 and ILF2 was minimized by Kaplan–Meier curves (**Figure 10H**) to compare the survival rates of the high- and low-risk groups at different times, and it was found that the survival rates of the low-risk group were higher, and the results were meaningful with *p* < 0.0001. The ROC curves (**Figure 10I**) showed that the values of the area under the curve (AUC) at the 1-year, 3-year, and 5-year periods were 0.72, 0.76, and 0.83, respectively, which indicated that the prediction models’ performance was stable and good. We further investigated the correlation between each prognostic gene and risk score (**Figure 10J**) and found that it was in line with our previous findings and visualized the top eight genes with strong correlation with risk score (**Figure 10K**). In addition, we explored the correlation between clinical factors such as age, gender, and ethnicity with risk scores (**Figures 11A–C**) and plotted nomograms based on risk scores versus clinical factors to predict the survival rates of different GBM patients at 1 year, 3 years, and 5 years (**Figure 11D**), which were validated using ROC curves and DCAs, and the ROC results showed that the AUC values of 1 year, 3 years, and 5 years were 0.69, 0.69, and 0.81 (**Figure 11E**), respectively, indicating that the sensitivity and specificity of the model were relatively good. The DCA (**Figure 11F**) also showed that the predictive ability of the new model was higher than that of the traditional model, indicating that the model we constructed has a better clinical predictive value.

### Immune correlation and enrichment analysis

**Figure 12:**
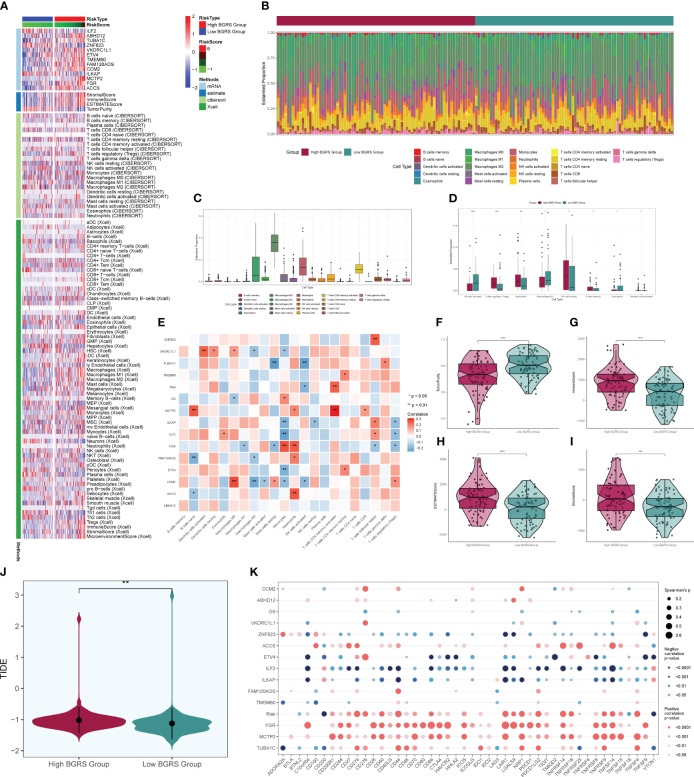
Immune correlation analysis. Heatmap of prognostic genes, tumor microenvironment, and immune cell situation in high- and low-risk groups. **(B)** Difference in immune cell ratio between high- and low-risk groups. **(C)** Difference in immune cell type scores. **(D)** Difference in immune cells between high- and low-risk groups. **(E)** Immunocell correlation with prognostic genes, risk scores, and OS. **(F–I)** Immune scores, stroma scores, tumor purity scores of tumor microenvironment of high- and low-risk groups, and the overall score difference plot. **(J)** TIDE score violin plot for high- and low-risk groups. **(K)** Degree of correlation between immune checkpoints and risk scores and prognostic genes. * means p < 0.05; ** means p < 0.01; *** means p < 0.001; **** means p < 0.0001.

**Figure 13:**
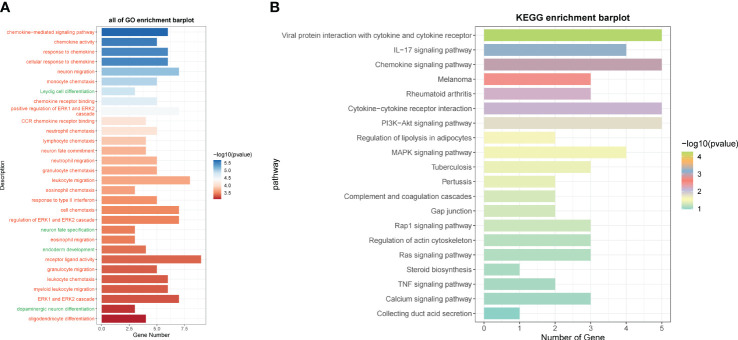
GO and KEGG enrichment analysis. **(A)** GO enrichment analysis of differential genes in high- and low-risk groups. **(B)** KEGG enrichment analysis of differential genes in high- and low-risk groups.

We calculated the immune cell infiltration in the high- and low-risk groups (**Figures 12A, B**) and further compared the immune cell differences between the high- and low-risk groups (**Figures 12C, D**), and found that macrophage M2 had the highest proportion, while M1 was highly expressed in the low-risk group, and we hypothesized that M1 might have good prognostic correlation. By correlation analysis of immunization with risk scores and prognostic genes (**Figure 12E**), we found that macrophage M1 was positively correlated with prognosis and negatively correlated with risk scores, which confirmed our speculation, whereas macrophage M0 was the opposite. We speculate that macrophage M1 and M0 may regulate tumor invasion and dividing ability in a non-directional manner and may pass through certain pathways of the transcription factor NFYB in order to fulfill related functions and interconvert each other. By comparing the tumor microenvironment-related scores in the high- and low-risk groups (**Figures 12F–I**), we found that the tumor purity score was higher in the low-risk group, whereas the immune score, stromal score, and total score were higher in the high-risk group. The tumor TIDE score (**Figure 12J**), on the other hand, showed a high score in the high-risk group, indicating the probability of immune escape in the high-risk group. We finally also calculated the immune checkpoint scores of the tumors and found that the low-risk genes were mostly negatively correlated with the immune checkpoints (**Figure 12K**), while the high-risk genes were mostly positively correlated, among which CD200 and VTCN1 were positively correlated with the low-risk genes and negatively correlated with the risk scores, so we presumed that they might have a certain inhibitory effect on the tumors, whereas CD44, LAIR1, etc. might have a certain protective effect on the tumors. By enrichment analysis, we found that in GO analysis (**Figure 13A**), the enriched pathways were mostly related to chemokines and immunity, such as “chemokine-mediated signaling pathway”, “regulation of ERK1 and ERK2 cascade”, and “leukocyte migration”, and in KEGG (**Figure 13B**), they were also associated with certain chemokines and signaling pathways, such as the “IL-17 signaling pathway”, “Chemokine signaling pathway”, and “Cytokine–cytokine receptor interaction”, and we hypothesize that the transcription factor NFYB may achieve certain pathway functions through the regulation of transcription factors to achieve certain promotion or inhibition of tumors.

### Experimental result/laboratory finding

**Figure 14:**
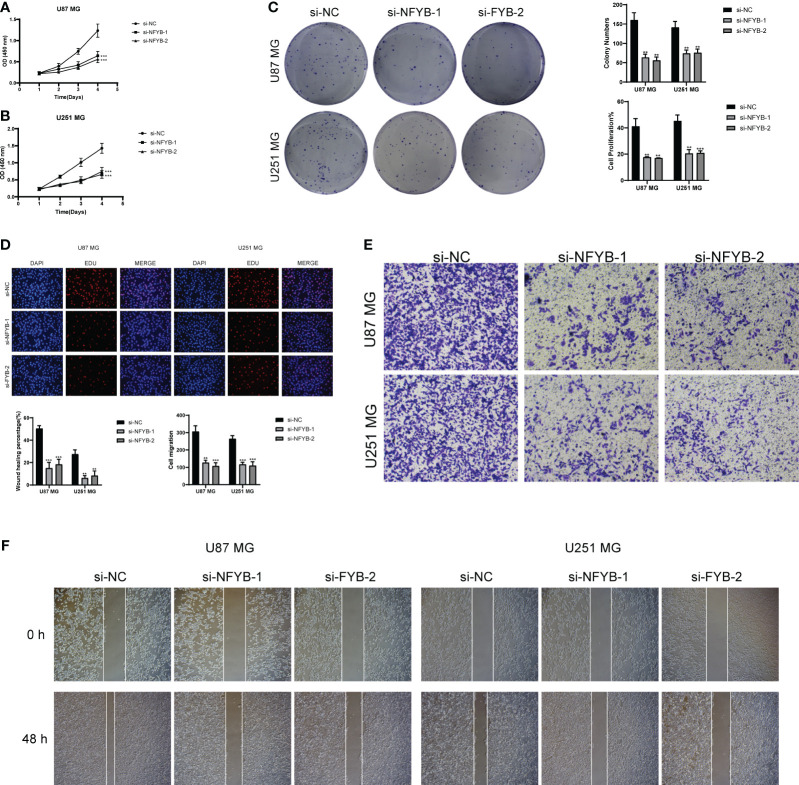
*In vitro* experimental validation of NFYB. **(A, B)** CCK-8 assay showed that cell viability was significantly reduced after NFYB knockdown. **(C)** Colony formation assay showed that the number of colonies in NFYB knockdown cells was significantly lower than that in the negative control group. **(D)** EdU staining showed that NFYB knockdown inhibited the proliferation of U87 MG and U251 MG cells. **(E)** Transwell assay revealed that NFYB knockdown significantly slowed down the invasion of U87 MG and U251 MG cells. **(F)** Scratch assay showed that NFYB knockdown significantly slowed down the migration of U87 MG and U251 MG cells. ** means p < 0.01; *** means p < 0.001.

We selected a total of two GBM cell lines for experiments, and compared the experiments by setting up a negative control group and a knockdown infection group. In the cell activity assay (**Figures 14A, B**), we found that CCK-8 assay showed a significant decrease in cell viability after NFYB knockdown. In the colony formation assay (**Figure 14C**), the number of colonies in BFYB knockdown cells was significantly lower than that in the negative control group. In the cell value-added assay experiment (**Figure 14D**), the cell numbers of both GBM cell lines with NFYB knockdown were lower than those of the negative control. In the Transwell assay experiment (**Figure 14E**), the staining areas of both cell lines with knocked-down NFYB were significantly smaller than those of the negative control. In the wound healing assay experiment (**Figure 14F**), the width of the 48-h scratch was significantly higher in both cell lines with knocked-down NFYB than in the negative control group. Therefore, through the experiment, we found that the proliferation, migration, and invasion ability of the U87 MG cell line and the U251 MG cell line with knocked-down NFYB were reduced, indicating that NFYB has a positive effect on the proliferation, migration, and invasion ability of GBM, and can promote the progression of GBM tumors.

## Discussion

GBM is a common malignant tumor of the nervous system, and the number of deaths due to GBM in the United States can reach approximately 15,000 per year (www.braintumor.org), and the average survival of GBM patients is only 14.6 months, even with adequate surgery and related radiotherapy and chemotherapy (8). Moreover, treated GBM patients are highly susceptible to recurrence, and even surgery, as the primary therapy, has been shown to cause tumor recurrence in preclinical models (55, 56), and recurrent GBM is often accompanied by high invasiveness and proliferation, as well as resistance to conventional therapy (57, 58). Therefore, we hope to search for the key mechanisms of GBM value-added and recurrence through single-cell analysis, in order to find new treatment directions and construct prognostic models.

By studying the various subgroups of GBM, we found that there were significantly more G2M-staged cells in the Glioma group, the distribution of which was concentrated in one of the Glioma subgroups, and the location was relatively overlapped with that of ndGBM and rGBM, which indicated that their dividing and value-added and invasive abilities might be stronger. In the GO and GSEA enrichment analysis of the Glioma subgroup, oxidative respiration-related pathways all scored high, such as oxidative phosphorylation, and correspondingly, Glioma immune-related pathways scored low. According to the relevant literature, oxidative phosphorylation occurs mainly in the mitochondria and plays a crucial role in some vital processes of tumors (59–62). Certain studies have shown that tumor stem cells with high metastatic and tumorigenic capacity are more dependent on oxidative phosphorylation (63, 64), and the process of oxidative phosphorylation also generates ROS, induces mutations, and suppresses the immune response, which has a tumor-promoting effect (65, 66). Garofano et al. found that GBM relies primarily on oxidative phosphorylation for energy and shows a marked vulnerability to inhibitors of its process (67), whereas Janiszewska et al. found that inhibition of IMP2 leads to impaired delivery of mRNA to the mitochondria and facilitates the assembly process of Complex I and Complex IV. This leads to an abnormal GBM oxidative phosphorylation process, which, in turn, impairs GBM tumor value-adding capacity (68). We found that enrichment analysis revealed a high enrichment of oxidative phosphorylation function in Glioma, while the key subgroup C5 was enriched in pathways related to cell division and proliferation capacity. Moreover, within the metabolic pathways, the oxidative phosphorylation score of C5 was notably significant, indicating a potential correlation between oxidative phosphorylation and the division and proliferation capacity of C5 subgroups, possibly contributing to tumor progression. Additionally, the ROS generated during oxidative phosphorylation can inhibit immune responses, induce the secretion of extracellular vesicles, and further enhance the production of IFN and IL-6 by macrophages, thereby suppressing immune responses in the tumor microenvironment (69–71). This finding aligns with our GSEA results, indicating a close association between the enriched pathways in the low-expression group and immune-related processes.

By studying the transcription factors of C5, a key subgroup with high dividing and proliferative capacity, we found that NFYB scored high in less relevant studies, which aroused our interest. NFYB is one of the subunits of NFY, which is a ubiquitous heterotrimeric transcription factor consisting of three subunits, namely, NFYA, NFYB, and NFYC, and can co-recognize the CCAAT box (72, 73). NF-YB contains histone motifs and can form dimers, which can drive the transcription of certain cell cycle-related genes, especially in the promoters of genes regulated in the G2M phase, suggesting that NF-YB can be involved in the regulation of cellular value-adding and apoptosis (74–77). Jiang et al. (78) found that knockdown of NFYB resulted in the downregulation of the apoptosis suppressor AIP5 and apoptosis inducer SIVA1 appeared upregulated, indicating that the presence of NFYB could promote cell division and value-added abilities, inhibit apoptosis, and promote tumor progression. More studies have shown that the high expression of NFYB is closely related to the elevation of tumor resistance to chemotherapeutic agents (79, 80). Our study also found that the C5 subgroup with high NFYB expression had the highest G2M score and a relatively higher proportion of rGBM, indicating that NFYB is highly associated with GBM proliferation and recurrence resistance, consistent with previous studies on NFYB in other cancers. Interestingly, NFYB also has a close relationship with mitochondria, not only participating in various mitochondrial-related signal transduction pathways, but also regulating mitochondrial function and activity through the NFYB-1–SPP-8 axis, which has a positive effect on extending mitochondrial lifespan (81). Moreover, we found that oxidative phosphorylation, a metabolic process that also occurs in mitochondria, has a certain promoting effect on GBM. Therefore, we speculate that NFYB may be related to oxidative phosphorylation, and that targeting NFYB can inhibit oxidative phosphorylation and mitochondrial respiration, thereby suppressing GBM progression, recurrence, and resistance. Through the prognostic analysis of NFYB transcriptional regulatory module genes and GBM differential genes, we finally identified 13 genes associated with prognosis, in which high-risk genes stood for the majority. Among them, TMEM60 was the gene with the highest risk score, which belongs to the transmembrane protein family (TMEM), and TMEMs are mostly involved in adverse events related to tumors, such as TMEM88 that leads to proliferation, migration, and invasion of gastric cancer tumors through the JAK2/STAT3 pathway (82). It has been previously reported that TMEM60 was closely associated with poor prognosis in GBM patients (83), but no correlation analysis was given, while Yang et al. (84) also found that high expression of TMEM60 would lead to poor prognosis in GBM patients and hypothesized that it might be related to immunosuppression. We found that TMEM60 belongs to the transcriptional regulatory module gene of NFYB, and realized that it may promote cell proliferation and drug resistance and lead to poor prognosis of GBM patients, and by targeting and regulating NFYB, we can make TMEM60 low expression to inhibit proliferation, invasion, and drug resistance of GBM cells, which will be beneficial to the prognosis of patients. Moreover, we found that the proliferation, migration, and invasion ability of GBM were greatly reduced by knocking down NFYB; thus, we hypothesized that NFYB is a high-risk transcription factor for GBM and also has the possibility to be used as a potential therapeutic target. However, the recurrence and resistance of NFYB in GBM, and the specific relationship and mechanism between NFYB and oxidation-related pathways still need to be verified by further experiments.

In summary, our study explored the specific mechanisms of GBM progression at the single-cell level, investigated the role of the transcription factor NF-YB and oxidative phosphorylation in GBM, and validated these findings through experiments. We also identified high-risk genes associated with prognosis. These studies will contribute to understanding the progression, recurrence, and drug resistance mechanisms of GBM, guiding new treatment strategies, identifying novel therapeutic targets, and providing new directions for the prognosis and diagnosis of GBM.

## Conclusion

In this study, we systematically explored the clinical subtypes of GBM and the associated mechanisms with the oxidative pathway using single-cell data. We identified the highly proliferative C5 subgroup and the transcription factor NFYB, which were subsequently validated through cell experiments. Our research confirms the significant role of transcription factors in GBM progression, providing new insights for future GBM studies. We discovered that targeting NFYB and the oxidative phosphorylation pathway could serve as potential key targets for GBM treatment and recurrence resistance. This finding opens new avenues for the treatment and prognostic diagnosis of GBM in the future.

## Data availability statement

The original contributions presented in the study are included in the article/**Supplementary Material**. Further inquiries can be directed to the corresponding authors.

## Ethics statement

The study protocols were performed according to Declaration of Helsinki.

## Author contributions

PL: Conceptualization, Data curation, Formal Analysis, Investigation, Methodology, Project administration, Resources, Software, Validation, Visualization, Writing – original draft, Writing – review & editing. NX: Conceptualization, Data curation, Project administration, Visualization, Writing – original draft. ZX: Conceptualization, Data curation, Formal Analysis, Methodology, Writing – original draft. JY: Resources, Software, Writing – original draft. ZL: Investigation, Resources, Supervision, Validation, Visualization, Writing – review & editing. JZ: Funding acquisition, Supervision, Writing – review & editing.

## Supplementary material

Supplementary Figure 1 Data quality control and dimensionality reduction clustering. **(A, B)** Quality control and filtered GBM single-cell related data. **(C)** Filtered GBM cell staging assay, including G1, G2M, and S stages. **(D)** Selected the top 2,000 highly variable genes based on gene expression and dispersion, indicating the top 10 highly variable genes. **(E)** Generated a PCA dimensionality reduction plot with the top 30 dimensions out of 50 selected. **(F)** PCA plots showcasing different sample sources after dimensionality reduction. **(G)** Heatmap of the top 10 highly variable genes in the first nine dimensions.
