# Supplementary figures and images for: Unraveling the intricacies of glioblastoma progression and recurrence: insights into the role of NFYB and oxidative phosphorylation at the single-cell level
Source: Front Immunol. 2024 Mar 6;15:1368685. doi: 10.3389/fimmu.2024.1368685 (PMC10950940; doi:10.3389/fimmu.2024.1368685)

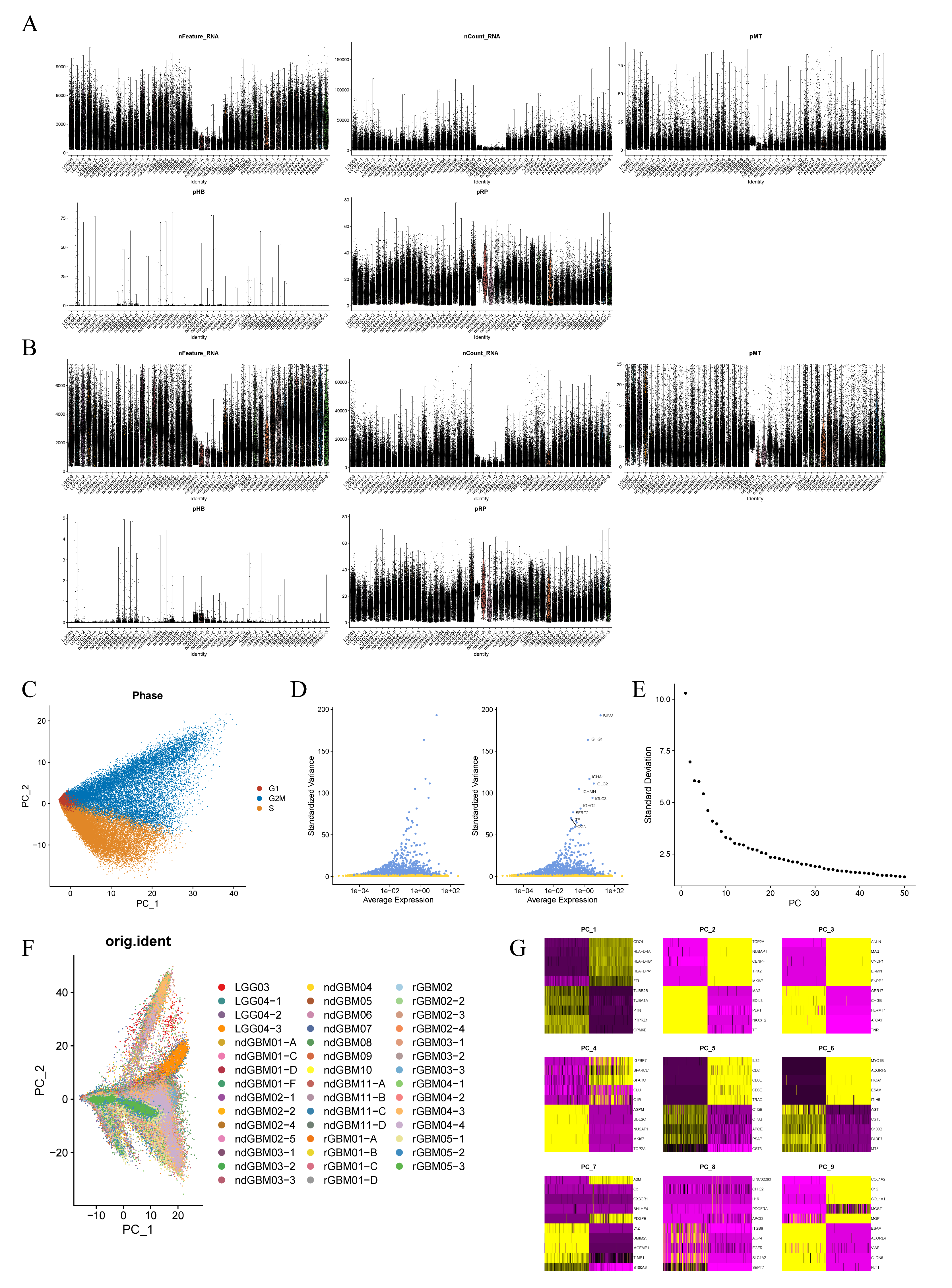

Supplement: Supplementary Figure 1 — Data quality control and dimensionality reduction clustering. (A, B) Quality control and filtered GBM single-cell related data. (C) Filtered GBM cell staging assay, including G1, G2M, and S stages. (D) Selected the top 2,000 highly variable genes based on gene expression and dispersion, indicating the top 10 highly variable genes. (E) Generated a PCA dimensionality reduction plot with the top 30 dimensions out of 50 selected. (F) PCA plots showcasing different sample sources after dimensionality reduction. (G) Heatmap of the top 10 highly variable genes in the first nine dimensions. [file Image_1.tif]
